# Investigation of Beeswax–Calcite Microcapsules as PCM for Latent Thermal Energy Storage in Building Applications

**DOI:** 10.3390/ma18245521

**Published:** 2025-12-09

**Authors:** Sameh Attia-Essaies, Houda Saad, Bochra Daghari, Rafika Ben Sghaier, Salwa Bouadila, Paulo Mira Mourão, Ezzedine Srasra

**Affiliations:** 1 Composite Materials and Clay Minerals Laboratory, National Center for Research in Materials Science, Technopole of Borj Cedria, Slimane 8027, Tunisia; saad_houda@yahoo.com (H.S.); daghariboshra.1@gmail.com (B.D.); rafika.bensghaier@fst.utm.tn (R.B.S.); srasra.ezzedine@gmail.com (E.S.); 2 Research and Technology Center of Energy, Hamam Lif 2050, Tunisia; salwa.bouadila@crten.rnrt.tn; 3 Department of Chemistry and Biochemistry, School of Science and Technology, University of Évora, 59 Romão Ramalho Street, 7000-671 Évora, Portugal; 4 MED—Mediterranean Institute for Agriculture, Environment and Development & CHANGE—Global Change and Sustainability Institute, University of Évora, Mitra Campus, P.O. Box 94, 7006-554 Évora, Portugal

**Keywords:** beeswax, calcite, vaterite, BW@CaCO_3_ microcapsules, thermal energy storage

## Abstract

Phase change materials (PCMs) are widely used for thermal energy storage; however, improving their thermal stability and minimizing supercooling effects remain important challenges. This study addresses these issues by synthesizing and characterizing new microencapsulated MCPs (microPCMs) that incorporate beeswax (BW), a sustainable biological source derived from animals, thus reducing the use of paraffins from petroleum resources, as the main material and calcium carbonate (CaCO_3_) as the shell to improve overall performance. MicroPCMs with variable shell contents (20%, 40%, 60%, and 80%) were prepared and analyzed using Fourier Transform Infrared Spectroscopy (FTIR), X-ray diffraction (XRD), Scanning Electron Microscopy (SEM), particle size distribution analysis (PES), and differential scanning calorimetry (DSC) to evaluate their structural, morphological, and thermal properties. The results reveal that microPCMs exhibit a spherical morphology and robust core–envelope integrity, with thermal energy storage capacities ranging from 121.39 to 122.22 J/g, compared to 137.62 J/g for pure beeswax. In addition, the composites demonstrated reduced supercooling and stable thermal performance during repeated cyclic tests. This work introduces the use of calcium carbonate shells combined with a natural beeswax core to create environmentally friendly microPCMs with enhanced thermal stability and reduced supercooling, offering a sustainable alternative for efficient thermal energy storage.

## 1. Introduction

In recent decades, the need for energy in its various forms has continuously increased due to the growth of industrial activities and the desire for convenience in daily life, including transportation, heating, and air conditioning [1]. Fossil fuels such as oil, coal, and natural gas currently meet the majority of global energy needs. However, this energy model faces two major constraints: environmental impact (such as climate change) and the depletion of fossil fuel resources [2]. To address these challenges, numerous studies have focused on exploring new sources and forms of energy production. Among renewable energies, solar radiation stands out, especially when coupled with thermal energy storage systems. Energy storage generally consists of accumulating energy when it is plentiful or less costly (for instance, solar energy captured during daytime or during off-peak hours for solar-drying applications [3] and shelters [4] so it can be used later during periods of limited availability or higher prices [5].

**Figure 1:**
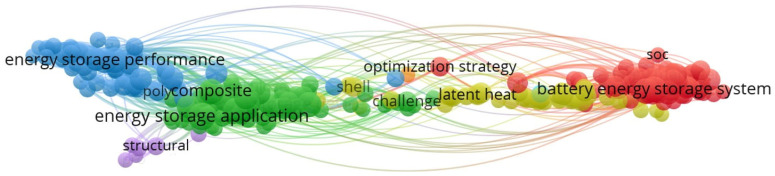
Research addressing thermal energy storage in the world.

Solar energy presents storage challenges due to its intermittent nature, being available only during daylight hours [6]. Therefore, it is essential to develop storage devices that can transfer energy from surplus to deficit periods [7], enabling the alignment of energy production with consumption patterns [8]. The prominence of thermal energy storage emerged in the 1980s following the 1973 oil crisis. Following this initial surge in interest, attention towards thermal energy storage diminished as oil prices declined. Energy storage is recognized as a major challenge of the 21st century [9]. Furthermore, the development of energy storage in its “thermal” form is seen as a tool that can enhance the competitiveness of various sectors and technologies: district heating and cooling networks, concentrated solar thermal power plants, the agri-food industry, housing, electronics, and more [10]. The bibliometric map provides a summary overview of the current trends in energy storage research based on 21,507 articles indexed in Scopus from the years 2024–2026 (Figure 1). The VOSviewer (version 1.6.18) co-occurrence network reveals five closely linked thematic clusters, ranging from performance assessment and development of applications to advances in composites and structural design. Other clusters highlight the ongoing efforts to optimize storage systems, in particular through strategies addressing operational problems and latent heat storage, as well as the rapid expansion of work on storage systems, including improved charging management. The strong interdisciplinary links in the field show the shift from basic materials research to integrated, system-level optimization and the emerging thermal and electrochemical storage solutions.

Thermal energy storage encompasses various systems for storing thermal energy in its different forms: “sensible,” “latent,” and “thermochemical” [11]. Sensible heat storage involves heat exchange without a phase transition, which is achieved by altering the temperature of the storage material [12]. Thermochemical storage relies on reversible endothermic/exothermic chemical reactions for energy exchange to enable storage [13]. In latent heat storage, the energy exchange leads to a phase change in the material (PCM) [14]. According to Kumar et al., PCMs have been gaining attention due to their substantial energy storage potential, which can be further enhanced through the incorporation of hybrid nanoparticles to boost thermal conductivity and overall performance. Ongoing developments in encapsulation methods and nanocomposite PCM engineering are significantly improving the efficiency of solar thermal energy storage and expanding its use in various low-temperature applications [15]. Due to their high latent heat of phase change, PCMs have the unique ability to absorb and release significant amounts of heat during phase transitions, typically solid–liquid shifts [16], at certain, near-constant temperatures [17]. This property allows PCMs to undergo phase changes as they absorb heat and release energy when needed [18]. The basic properties of a PCM include its phase change temperature and its latent heat [19]. PCMs are attracting significant attention and usage for several reasons. They are environmentally friendly, recyclable [20], non-toxic [21], non-corrosive, non-explosive, and compatible with various materials [20]. They exhibit high thermal capacity [22], long-term chemical stability, favorable chemical properties, and can undergo freeze/thaw cycles. These materials exhibit non-flammable characteristics and demonstrate long-term thermal cycling stability without observable decomposition [20]. These materials can undergo diverse transformations [23]. PCMs can be utilized for solid–solid [24], solid–liquid [25], or liquid–gas transitions [26]. Solid–gas and liquid–gas transitions are impractical due to system volume and pressure concerns [27].

There are numerous factors influencing the efficiency and applications of solid–liquid PCMs: thermal capacity, thermal conductivity [28], latent heat [29], phase transition temperature [30], and other factors [31]. Solid–liquid PCMs demonstrate a good balance between storage capacity and manageable phase transition characteristics while maintaining practical implementation requirements [28], making them ideal candidates for advanced thermal energy storage systems [32].

Thermal storage utilizing latent heat represents a promising approach from both environmental and energy efficiency standpoints [33]. The advantage of PCMs lies in their capability to store a substantial amount of energy while maintaining a favorable mass/volume ratio. However, most PCMs exhibit relatively low thermal conductivity (0.1–0.4 W/mK), which hinders efficient heat transfer and can lead to leakage during the melting or solidification process, limiting their practical applications [34]. PCMs are the materials utilized in this method, known for their ability to transition between different physical states within specific temperature ranges, with melting/solidification being the most common phase change within these intervals [35]. PCMs exhibit a high heat capacity within a restricted temperature range, making them suitable for near-isothermal heat storage [36].

The categorization of PCMs includes organic [37], inorganic, and eutectic types [38]. Among organic PCMs, they can be further delineated into paraffinic (e.g., petroleum waxes and beeswax) and non-paraffinic materials such as fatty acids, alcohols, glycols, esters, and polyols. Organic compounds typically have low densities (0.7 to 1.6), melting temperatures below 150 °C, and thermal conductivity around 0.2 W/mK, with optimal candidates having phase change enthalpies between 250 and 300 J/g [10]. Paraffins, sourced from plants, animals, minerals, and petroleum, constitute organic PCMs [39]. They are also non-toxic, thermally stable, and offer a wide range of phase change temperatures up to 120 °C [40]. Beeswax results from the metabolic processes of bees in hives and is released (excreted) through the bees’ abdominal segments [41]. Two types of beeswax are distinguished: yellow beeswax, which has a honey aroma but is brittle in the solid phase, and white beeswax, which lacks a honey aroma but is more flexible than yellow beeswax. White beeswax cannot dissolve in water, but it can easily dissolve in chloroform. Beeswax mainly consists of esters, fatty acids, and long-chain alcohols, with an empirical formula of C_15_H_31_COOC_30_H_61_, classified as organic PCMs [42].

Addressing these challenges requires technology capable of containing PCMs in either liquid or solid states within a solid enclosure to isolate them from the external environment. This technology, known as encapsulation, not only helps retain PCMs but also enhances heat transfer efficiency, prevents PCM leakage after repeated thermal cycles, and enables the materials to withstand frequent volume changes during phase transitions. To overcome these issues, encapsulated MCPs have been developed, employing a process of individual coating for MCP droplets with a continuous film to produce encapsulated MCPs, offering reduced undercooling, a large heat transfer area, and control over volume change during phase transition [28]. Encapsulation, which involves confining the required material within another envelope material, is crucial for achieving desired preservation characteristics, controlled material release over time, environmental reaction reduction, and corrosion prevention [43]. Encapsulation emerges as an intriguing alternative for various purposes including protecting and stabilizing active principles, handling toxic substances, simplifying viscous liquid manipulation, preventing component interaction, masking undesirable odors or tastes, reducing chemical sublimation rates, controlling active principal release profiles, and drug vectorization [44]. The most common types of encapsulations include macroencapsulation, nanoencapsulation, and microencapsulation, with microencapsulation being the focus of this work [45]. Materials used for encapsulation play a vital role in morphological, mechanical, and thermal properties, with organic materials like melamine–formaldehyde resin, urea–formaldehyde resin, and acrylic resin offering good compactness and structural stability but exhibiting limitations such as flammability, low thermal conductivity, mechanical strength, and chemical stability. In contrast, inorganic shell materials like zinc oxide, titanium dioxide, silica, and calcium carbonate provide higher rigidity, mechanical strength, and thermal conductivity, making them promising choices for enhancing microencapsulated MCP performance [46]. Among these, calcium carbonate has gained considerable interest for its widespread applications, various crystallographic forms, stability, and affordability, with studies focusing on microcapsules utilizing calcium carbonate shells demonstrating improved thermal conductivity and durability [47].

Microencapsulated phase change materials based on beeswax are a promising class of thermal energy storage systems made from renewable and natural resources. Beeswax’s high latent heat of fusion and favorable thermal stability allow for effective heat absorption and release during phase transitions [48]. Beeswax is a desirable option for microPCM development aimed at consumer goods, textiles, and construction because of its inherent qualities. Innovative thermal energy storage solutions called beeswax-based microencapsulated phase change materials (microPCMs) make use of beeswax’s inherent qualities to improve energy efficiency in a variety of applications [49]. It should be noted that the melting temperature range of 55–62 °C for the beeswax is selected based on medium-temperature thermal energy storage applications. This range allows the PCM to meet under moderate heating conditions, ensuring efficient charging and discharging without requiring excessive energy input. Beeswax naturally exhibits a melting interval rather than a single point because of its complex composition of esters, fatty acids, and hydrocarbons. Choosing a PCM within this range ensures consistent thermal behavior and good phase stability. The complex mixture of esters, hydrocarbons, and fatty acids that make up beeswax, which is derived from the secretions of honeybees, contributes to its high latent heat of fusion and thermal stability. The incorporation of beeswax into functional composite systems has been the subject of numerous studies. Brito-Pereira et al. [50] showed that natural beeswax reinforced with conductive nanofillers like graphene can produce multifunctional materials with high conductivity, intrinsic recyclability, and notable piezoresistive and thermoresistive sensing capabilities, supporting solvent-free additive manufacturing for flexible electronics and biomedical applications. Alvarez-Gallardo et al. [51] incorporated beeswax directly into bitumen/SBS blends to produce form-stable roofing materials containing up to 30 wt% beeswax, obtaining improved thermal storage capabilities and a quantifiable decrease in the need for indoor cooling. Additionally, beeswax and polyethylene glycol have been combined to produce eutectic organic PCMs that have better chemical compatibility and adjustable melting behavior, as shown by Sharma et al. [52]. In building applications, Medjahed et al. [53] reported that increasing the content of beeswax and paraffin PCMs in plaster composites can significantly decrease thermal conductivity and diffusivity, improving indoor comfort through enhanced insulation and latent heat storage. The rising demand for sustainable materials further underscores the appeal of beeswax-based microPCMs as environmentally friendly alternatives to conventional synthetic PCMs. Beyond beeswax systems, shape-stabilized PCMs (SSPCMs) provide another strategy for overcoming leakage and improving thermal conductivity. Despite their advantages, challenges remain for beeswax-based microPCMs. As discussed by Chinnasam et al., issues such as high production costs, variability in natural raw material supply, and limited market awareness may impede their competitiveness with petroleum-derived counterparts. Achieving performance comparable to traditional microPCMs continues to require targeted research and technological development to ensure consistent quality and functionality [54].

**Figure 2:**
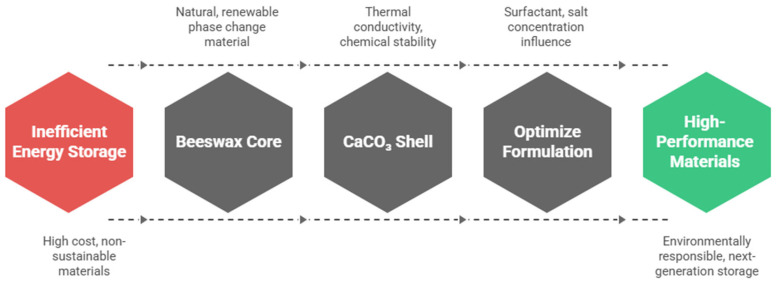
Study of beeswax/calcium carbonate microcapsules for thermal energy storage.

The objective of this research is to design and study an innovative microencapsulation system that combines a natural, renewable beeswax-based phase change biomaterial with an inorganic calcium carbonate (CaCO_3_) shell. This work seeks to establish a sustainable and low-cost approach to thermal energy storage by exploiting the biodegradability, non-toxicity, and availability of beeswax, together with the thermal conductivity and chemical stability of CaCO_3_. Beyond the basic fabrication of microcapsules, the study aims to advance understanding of how formulation parameters, particularly surfactant choice and precursor salt concentrations, govern the structural integrity, thermal behavior, and encapsulation efficiency of microPCM. By exploring this hybrid organic/inorganic system, the work contributes an original pathway for developing environmentally responsible, high-performance materials for next-generation thermal energy storage applications. Figure 2 illustrates the layout of this study on beeswax/calcium carbonate microcapsules designed for thermal energy storage.

## 2. Materials and Methods

### 2.1. Materials

The beeswax used in this work has melting temperatures ranging from 55 °C to 62 °C. This beeswax is purchased from ISO 9001 [55] (Wax Foundation of Bees ELMaleka-certified company ISO 22000, 2005, Kraków, Poland) [56]. Sodium dodecyl sulfate (SDS) was used as an emulsifier. Calcium chloride (CaCl_2_), sodium carbonate (Na_2_CO_3_), and sodium dodecyl sulfate (SDS) (99% purity) were obtained from Sigma Aldrich (St. Louis, MO, USA).

### 2.2. Preparation of BW@CaCO_3_

**Figure 3:**
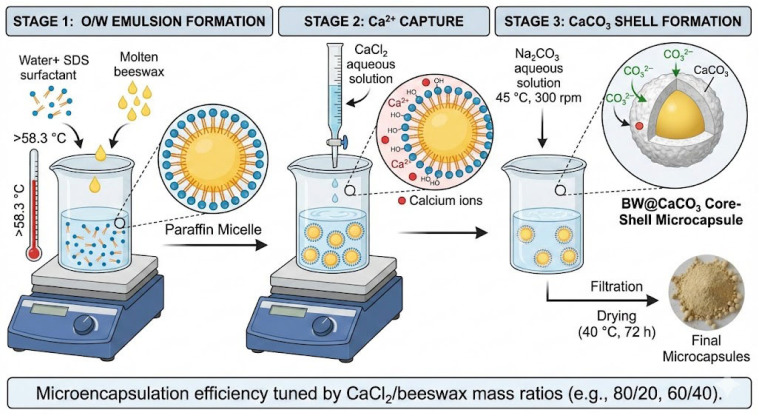
Schematic illustration of BW@CaCO_3_ preparation.

Microencapsulation was performed through spontaneous CaCO_3_ deposition onto beeswax droplets, resulting in core–shell structures (BW@CaCO_3_) with beeswax as the PCM core and calcite as the protective shell. The synthesis process of BW@CaCO_3_ microcapsules is straightforward, as illustrated in Figure 3. The polymerization process was conducted in three distinct stages, involving the preparation of an O/W emulsion, the creation of a prepolymer solution, and the production of microcapsules. Initially, 4 g of beeswax was melted at a temperature above its melting point (≈58.3 °C) and then placed in a 50 mL aqueous solution containing non-ionic surfactant SDS (0.5 g) in deionized water. This mixture was stirred for 20 min to establish a stable O/W emulsion system. During this phase, the hydrophobic chains of the surfactants were oriented towards the oil droplets. At the same time, their hydrophilic segments (such as hydroxyl groups) interacted with water molecules away from the oil phase. The surfactant layer neatly covered the surfaces of the oil droplets, forming paraffin micelles. Subsequently, CaCl_2_ (5.55 g, 0.05 mol) was solubilized in 75 mL of deionized water and added dropwise into the flask. After stirring for 3 h, self-assembly was initiated by slowly introducing a solution of Na_2_CO_3_ (5.3 g, 0.05 mol) dissolved in 75 mL of deionized water into the mixture under vigorous agitation at 300 rpm, a process that was maintained at 45 °C. The resulting microcapsules were recovered by filtration and dried at 40 °C for 72 h. Various CaCl_2_/beeswax mass ratios (80/20, 60/40, 40/60, and 20/80) were investigated to evaluate their influence on the microencapsulation efficiency. Changes in these ratios can affect the characteristics of the microcapsules, including shell thickness, encapsulation efficiency, and release properties. By fine-tuning this ratio, researchers can customize the microencapsulation procedure to achieve specific goals related to shell structure, stability, and functionality. To ensure the reliability and statistical validity of the results, all microcapsule synthesis experiments were performed in triplicate (*n* = 3).

The interaction between Ca^2+^ and the hydroxyl groups of the surfactants led to Ca^2+^ being captured during the addition of CaCl_2_ aqueous solution dropwise into the emulsion system. Ultimately, CaCO_3_ was formed through a precipitation reaction upon adding the Na_2_CO_3_ aqueous solution. Consequently, a CaCO_3_ shell was successfully synthesized onto the surface of the paraffin micelle through this self-assembly process [47].

### 2.3. Characterization

The morphology and chemical composition of microPCMs were investigated using Scanning Electron Microscopy (SEM) and the energy-dispersive spectrometer (EDS) through a JEOL JSM 6335F microscope (Tokyo, Japan).

The structural compositions of (microPCMs) were analyzed by a Nicolet Fourier Transform Infrared Spectrometer (FTIR, model 560, Waltham, MA, USA). The spectra of samples were obtained using attenuated total reflectance mode with a diamond crystal, ranging from 4000 to 400 cm^−1^.

The crystalline structure of the samples was analyzed using X-ray diffraction (XRD) over a 2θ range of 20° to 80° at a scanning rate of 2°/min. Measurements were performed with a Panalytical X’Pert diffractometer equipped with CuKα_1_ radiation, operating at 30 mA and 40 kV. The resulting diffractograms were subsequently processed using X’Pert HighScore Plus software (version 3.0.0.).

The particle size distribution (PSD) and the mean diameters of the microcapsules were determined using a Microstrac S3500 particle size analyzer (Haan, Germany).

The phase change temperature and the latent heat of the samples were measured by using a differential scanning calorimeter DSC Mettler Toledo 823 (Greifensee, Switzerland). Calibration was performed with indium in the temperature range of 0 to 350 °C. The sample weight was approximately 6.0–6.4 mg. Small masses were used to reduce the effects of side reactions as well as to limit mass and heat transfer. Experiments were conducted under nitrogen gas (N_2_) and a flow rate of 50 mL/min, from 20 to 500 °C at a heating rate of 10 °C/min.

## 3. Results and Discussion

### 3.1. Structural Properties of BW@CaCO_3_

#### 3.1.1. X-Ray Diffraction Analysis of BW@CaCO_3_

**Figure 4:**
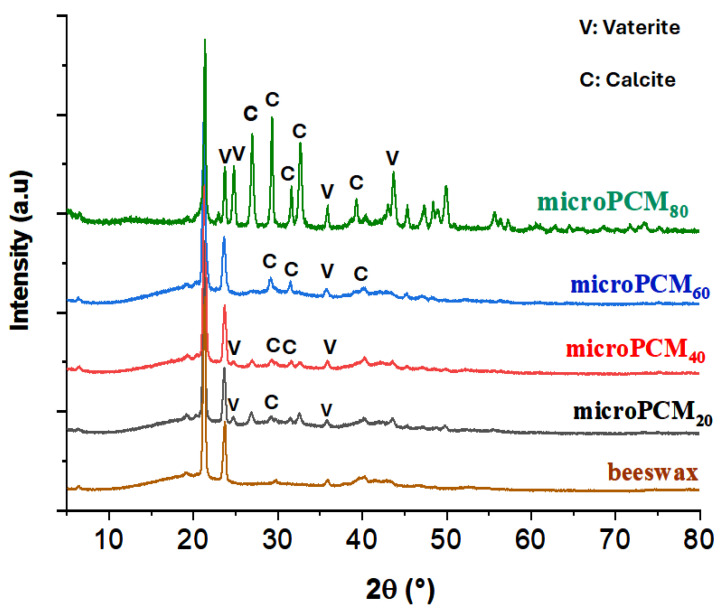
XRD spectra of BW and BW@CaCO_3_.

To enhance the structural investigation of beewax@CaCO_3_ microcapsules, X-ray diffraction (XRD) analyses were conducted. Figure 4 illustrates the diffractograms of pure beeswax and beeswax@CaCO_3_ microcapsules, along with reference samples of calcite CaCO_3_ (JCPDS Cards, 01-081-2027) and vaterite CaCO_3_ (JCPDS Cards, 01-074-1867). The distinctive diffraction peaks of beeswax at 2θ values of 21.34° and 23.79° correspond to interplanar spacings d_hkl_ of 4.16 Å and 3.74 Å, respectively. It is noteworthy that diffraction peaks at 2θ = 6.27°, 9.41°, 12.54°, and 19.17° are assigned to the (002), (003), (004), and (010) planes of paraffin, respectively.

A series of characteristic reflections at 2θ = 24.78°, 26.96°, and 32.62° linked to lattice plane values (110), (101), and (102) represent vaterite, respectively. Peaks at 2θ = 23.06°, 29.41°, 39.42°, and 47.53° are identified as the (012), (104), (113), and (018) planes of calcite CaCO_3_, respectively [57]. The emergence of diffraction lines at angles 2θ = 29.52°, 31.88°, and 35.72°, attributed to lattice plane values (104), (006), and (110), are distinctive features of calcite.

The presence of unchanged beeswax diffraction lines and the coexistence of characteristic diffraction peaks of both CaCO_3_ polymorphs, vaterite and calcite, suggest weak interactions between the core (beeswax) and the calcium carbonate CaCO_3_ (shell), supporting the composition of the shell as a combination of calcite and vaterite, as evidenced by the coexistence of characteristic lines from both polymorphs in the beeswax spectra.

#### 3.1.2. FTIR Analysis of BW@CaCO_3_

**Figure 5:**
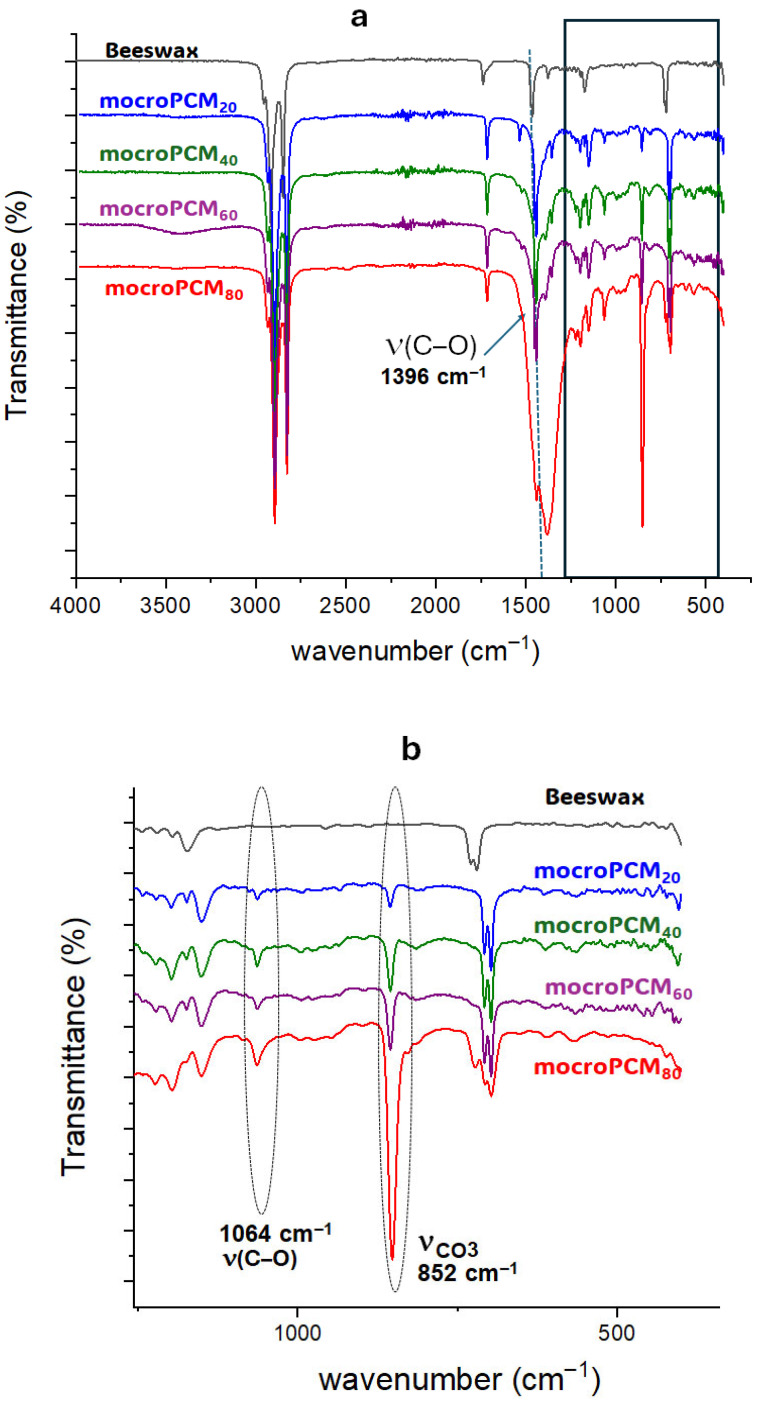
FTIR spectra of (**a**) BW and BW@CaCO_3_; (**b**) enlarged view of the 1250–400 cm^−1^ region.

Figure 5 depicts the FTIR spectrum of BW, calcite, and the BW@CaCO_3_ composite. The bands at 2932 cm^−1^ and 2850 cm^−1^ signify the elongation vibration of groups -CH_2_ and -CH_3_ in the BW spectrum, while the peak at 731 cm^−1^ corresponds to the vibration in the group plane (CH_2_)_n_. Furthermore, the characteristic peak at 1396 cm^−1^ corresponds to the asymmetric stretching vibration of CO_3_^2−^. The spectrum of BW@CaCO_3_ shows absorption peaking at 884 cm^−1^, with 1069 cm^−1^ assigned to the bending vibration in the plane of C-O-C in calcite and vaterite, respectively [58]. The FTIR spectra of composites indicate the characteristic peaks of beeswax and CaCO_3_, suggesting the presence of both in the microcapsules. The coexistence of vaterite and calcite polymorphs arises from nucleation and growth conditions characteristic of the self-assembly process, wherein molecules or particles spontaneously organize into ordered structures driven by physicochemical interactions. These interactions are modulated by the types of emulsifiers and the concentrations of Ca^2+^ and CO_3_^2−^ ions. The choice of emulsifier plays a pivotal role in determining the crystalline phase of the CaCO_3_ shell. During precipitation, phase transformations may occur, leading to the formation of calcite, vaterite, or their coexistence. Anionic emulsifiers such as SDS used in this study can selectively adsorb onto specific crystal faces, thereby influencing nucleation pathways and controlling the final morphology of the shell. XRD analysis confirms the simultaneous presence of vaterite and calcite, indicating that CaCO_3_ particles assemble around beeswax through adsorption, electrostatic interactions, and van der Waals forces, without undergoing chemical reaction. Moreover, the increasing intensity of the characteristic calcite band observed with the rise in the mass ratio (CaCl_2_/beeswax) suggests that the amount of precursors (CaCl_2_ and Na_2_CO_3_) directly influences the formation and thickness of the mineral shell. Consequently, the original crystalline structures of the polymorphs remain intact, and CaCO_3_ functions as a protective shell, preserving the chemical integrity of beeswax while enhancing the mechanical and thermal stability of the composite.

#### 3.1.3. Particle Size Distribution of BW@CaCO_3_

**Figure 6:**
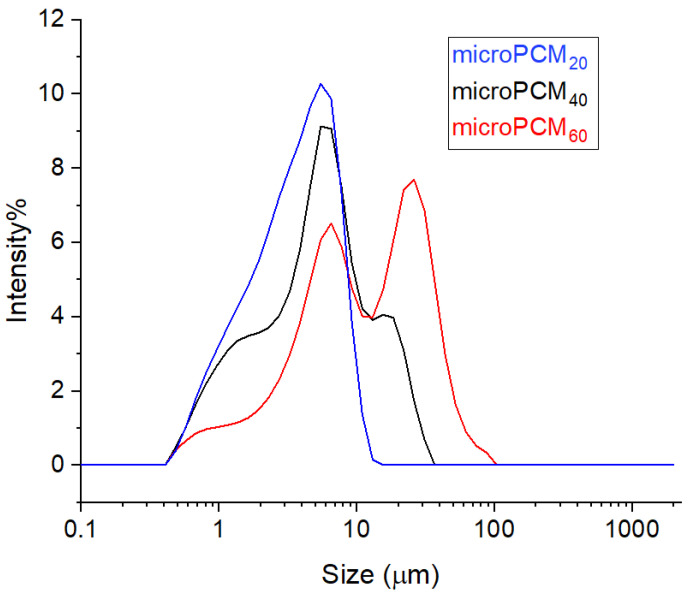
Particle size distribution images of BW@CaCO_3_ phase change microcapsules.

The curve particle size distribution (PSD) for the three microPCM samples, shown in Figure 6, clearly highlights the influence of the encapsulation process on particle size. The microPCM_20_ sample exhibits a unimodal and narrow distribution, with an average particle diameter of 3.72 μm and sizes ranging from 1.027 μm to 7.88 μm, indicating a homogeneous population. For microPCM_40_, the distribution becomes slightly broader, and the mean diameter increases to 6.47 μm, reflecting particle growth. At the highest concentration (microPCM_60_), the curve extends markedly towards larger particle sizes, reaching nearly 40 μm, and displays a bimodal trend, revealing two distinct populations. This progression confirms that increasing CaCl_2_ content promotes particle growth and heterogeneity during shell formation.

This observation demonstrates that calcite formation (CaCO_3_) within SDS-emulsified microcapsules can be linked to the tridentate coordination motif of oxygen atoms in the HSO_3_^−^ terminated monolayer. This structural arrangement facilitates the nucleation of carbonate ions aligned with the (001) crystal plane of calcite, stabilizing its growth [59]. Moreover, the influence of SDS on calcium carbonate crystallization is mainly related to its role as an anionic surfactant, capable of modifying nucleation kinetics and crystalline morphology. The SDS, consisting of a sulfated polar head and a long hydrophobic chain, selectively adsorbs on the crystalline faces of calcite, reducing their surface energy and inhibiting their growth. This adsorption disrupts the arrangement of Ca^2+^ and CO_3_^2−^ ions at the solid–liquid interface, which slows down the transformation to the thermodynamically stable form (calcite) and promotes the formation of metastable polymorphs such as vaterite.

#### 3.1.4. Morphological and Elemental Analysis of BW@CaCO_3_

**Figure 7:**
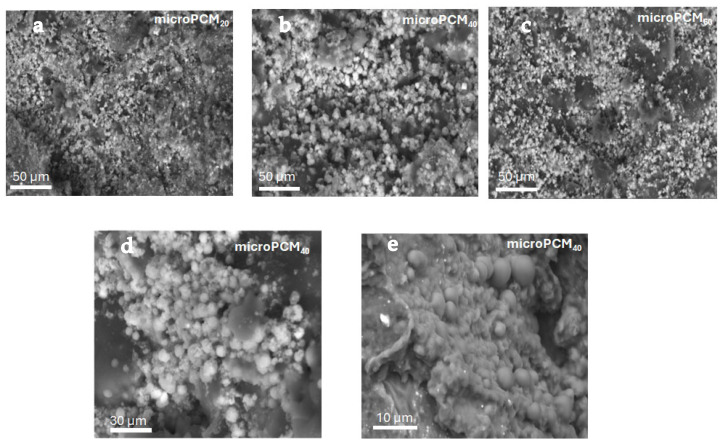
SEM of microcapsule PCMs with different weight ratios of beeswax/CaCl_2_ (**a**) microPCM_20_ 50 µm (**b**) microPCM_40_ 50 µm (**c**) microPCM_60_ 50 µm (**d**) microPCM_40_ 30 µm (**e**) microPCM_40_ 10 µm.

Figure 7 shows the morphologies examined using Scanning Electron Microscopy (SEM). It can be observed that all samples mostly have sphere-like morphologies with a variable average particle size in agreement with the results achieved by the previous particle size distribution studies.

It is common knowledge that CaCO_3_ has three crystalline polymorphs: calcite, vaterite, and aragonite. All of them have distinct morphologies and are appropriate for different uses. These phenomena suggest that the binary weight ratio of the core materials is a key factor influencing the crystalline form of CaCO_3_, which in turn results in various CaCO_3_ crystalline phases and different morphologies of microencapsulated phase change materials (microPCMs). However, some microcapsules tend to adhere to each other and form aggregates with a uniform internal structure and a smooth, dense surface. This aggregation leads to a wider particle size distribution than initially expected, as the clusters effectively increase the overall particle size beyond the expected range, which is consistent, once more, with the results of the particle size distribution.

**Figure 8:**
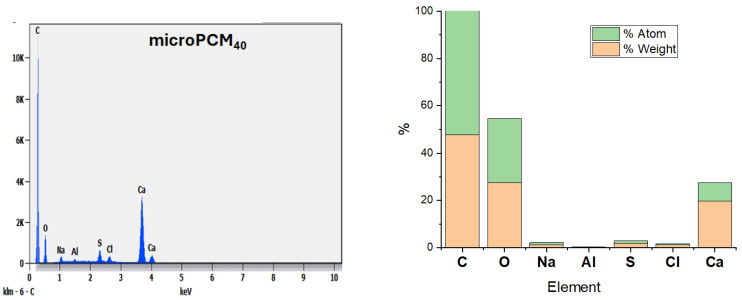
EDS image of microPCM_40_.

Figure 8 reveals that the microPCM_40_ mainly characterizes the CaCO_3_ shell of the microPCMs. From the EDS analysis data of microPCM_40_, it is evident that the synthesized microPCMs contained Ca, O, and C elements from the CaCO_3_ shell.

### 3.2. Thermal Performance of BW@CaCO_3_

**Figure 9:**
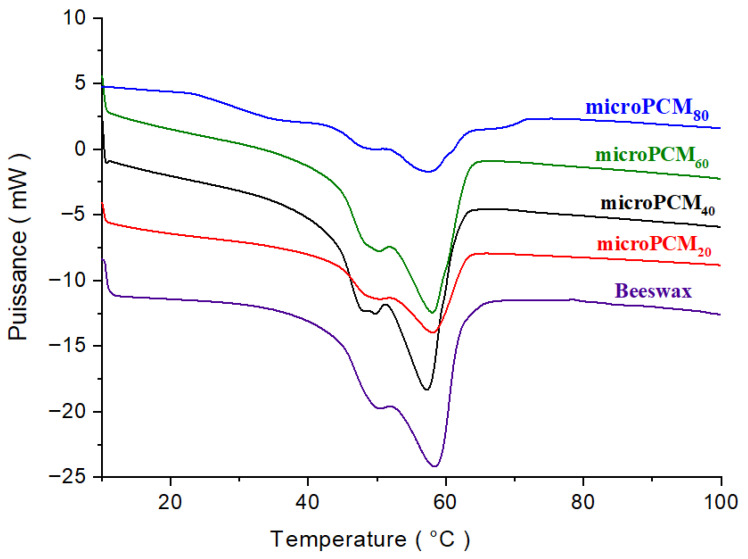
DSC curves of the BW and BW@CaCO_3_.

The impact of varying beeswax-to-CaCl_2_ weight ratios (80/20, 60/40, 40/60, and 20/80) was assessed in terms of both encapsulation efficiency and thermal behavior of the resulting microcapsules. Further information on the thermal properties of the BW@CaCO_3_ composite is given in Figure 9, which shows the DSC analysis results for the pure beeswax PCM. Phase change temperatures, enthalpies of pure beeswax, and those of microencapsulated beeswax are derived from the DSC profiles and summarized in Table 1.

A large peak on the DSC curve indicates that the beeswax is melting from a solid to a liquid, and a smaller peak may also show that the solid-to-solid transition has changed. The phase change peaks of beeswax are retained in shape-stabilized phase change composites, given that there were no chemical reactions between the beeswax and CaCO_3_ during their preparation.

The thermogram shows that the beeswax started to melt at a starting temperature of 38.92 °C. The beeswax (PCM) continued to melt until the temperature peaked at 58.30 °C. At the end temperature of 70 °C, the beeswax had completely melted. The area can be calculated based on the start, peak, and end temperature data. The latent heat of beeswax was calculated by dividing the area by the amount of sample used. The latent heat of beeswax was found to be 137.62 J/g. This value is comparable to the work of Fang et al. [39]. This value is determined by using numerical integration calculation by the DSC software Pyris (version 13.2.1.0007).

However, the encapsulation of beeswax with a CaCO_3_ shell significantly reduced the absolute phase change enthalpies of the microcapsule samples. Due to the inert CaCO_3_ shell, the latent heat can only be stored by the phase-changeable beeswax core. Therefore, the microcapsule samples’ phase change enthalpies strongly depend on the loading of beeswax inside the microcapsules. In the microencapsulation process, the encapsulation rate (Er) is crucial for the performance of the microPCM composites. It is defined as the ratio of the core mass (beeswax) to the shell mass (CaCO_3_), but measuring these masses directly can be challenging. For this reason, the encapsulation ratio (Er) and the theoretical enthalpy were calculated using Equations (1) and (2), respectively [47]. Equation (2) provides an estimate of the theoretical latent heat of microPCMs based on the proportions of precursors. This theoretical value serves as a reference and is generally compared to the experimental latent heat measured by DSC. Such a comparison is essential to evaluating the encapsulation efficiency and overall thermal performance of microcapsules.

(1) $$Encapsulation\;rate\;Er\left(\%\right)=\frac{∆Hf\left(microPCM\right)}{∆Hf\left(beeswax\right)}\times 100$$

(2) $${∆Hf\left(microPCM\right)}_{calculated}=\frac{∆Hf\left(beeswax\right)}{m\left(beeswax\right)+m\left({CaCl}_{2}\right)}\times m\left(beeswax\right)$$

where $∆Hf\left(beeswax\right)$ and $∆Hf\left(microPCM\right)$ are the latent heat of beeswax and microPCMs, respectively.

The DSC results in Table 1 show that the melting temperatures of the microencapsulated composites decrease by 0.24 to 1.81 °C when compared with the melting and solidifying temperatures of the pure beeswax (58.30 °C). This slight thermal shift is characteristic of encapsulation phenomena and is primarily attributed to the confinement effect. The rigid CaCO_3_ shell imposes spatial constraints on the beeswax core, restricting the size of the PCM crystalline domains. This reduction in crystallite size and the associated increase in surface area lead to the observed melting point depression, consistent with the Gibbs–Thomson effect. Furthermore, the CaCO_3_ shell may introduce interfacial interactions that slightly modify the crystallization kinetics of the core. Importantly, this minor thermal shift does not compromise the energy storage capability and may, in fact, enhance the thermal cycling stability by influencing the solid–liquid interface dynamics.

When comparing the experimental and calculated enthalpies of fusion and encapsulation rate, a notable discrepancy is observed. For instance, at a beeswax/CaCl_2_ ratio of 20/80, the experimental encapsulation rate was 83.43%, whereas the calculated value was only 59.81%. Since the experimental value is derived solely from the latent heat contribution of the PCM, this divergence indicates that the actual mass of the CaCO_3_ shell retained in the final microcapsules is significantly lower than the theoretical mass predicted from the initial precursor ratio. This reduction in shell mass is most likely due to incomplete precipitation of CaCO_3_ precursors and the subsequent loss of unbound particles during purification. Furthermore, this deviation can also be attributed to the agglomeration of CaCO_3_ particles during the in situ formation of the shell, which is not accounted for in the theoretical model. Such agglomeration reduces the available surface area for uniform shell formation, thereby affecting the encapsulation efficiency and heat storage capacity. To minimize CaCO_3_ particle agglomeration during in situ shell formation, a combination of chemical strategies (surfactants and stabilizers), physical methods (adequate stirring and ultrasonication), and kinetic control (pH adjustment and regulated precipitation rate) is recommended to achieve a uniform and well-dispersed shell structure. In addition, since the experimental determination is based exclusively on the PCM’s latent heat contribution, this divergence suggests that the actual mass of the CaCO_3_ shell retained in the final microcapsules is substantially lower than the theoretical mass derived from the initial precursor ratio. This shell mass reduction is likely attributed to the incomplete precipitation of CaCO_3_ precursors. Consequently, this leads to a thinner shell, which unexpectedly enhances the final product’s thermal performance.

**Figure 10:**
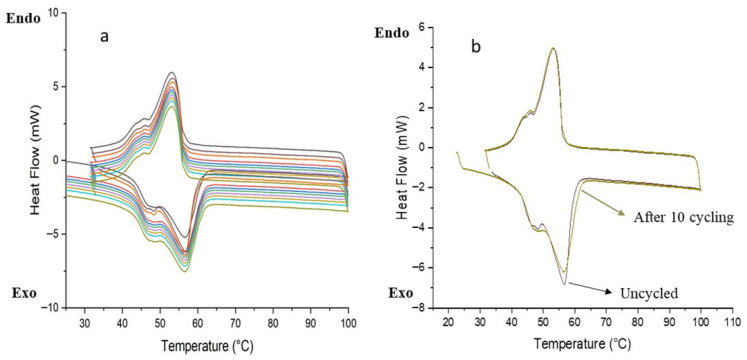
(**a**) DSC curves of microPCM_40_ after 10 thermal cycles; (**b**) DSC curves of microPCM_40_ before and after 10 thermal cycles.

Thermal reliability and reusability are essential characteristics for phase change materials (PCMs) intended for thermal energy storage systems. These materials must sustain consistent thermal properties across multiple phase transition cycles. Among the various formulations examined, the microPCM_40_ sample exhibited a favorable compromise between high encapsulation efficiency (88.93%) and stable thermal behavior. This composition was therefore selected for thermal cycling analysis. As illustrated in Figure 10, the differential scanning calorimetry (DSC) profiles recorded after ten successive melting–freezing cycles revealed negligible shifts in both the phase transition temperature and latent heat. These findings demonstrate that the sample retains its thermal performance upon repeated use. Based on the overall results, the composition corresponding to a 40% shell content was identified as the most suitable in terms of both calcite content and core–shell ratio.

These findings can be attributed to the synergistic effect of the coexistence of calcites and vaterites within the CaCO_3_ shell, which establishes an optimal balance of thermal properties. Vaterite, characterized by its porous and defect-rich structure, facilitates higher PCM loading and offers numerous nucleation sites, thereby enhancing latent heat storage and mitigating supercooling. In contrast, calcite, with its compact and thermodynamically stable structure, reinforces the shell and ensures superior cycling durability. The combination of these two polymorphs endows the microPCMs with both high thermal performance and long-term structural stability. Furthermore, microcapsules exhibit a predominantly spherical morphology with micrometer-scale diameters, which improves their processability and broadens their potential for integration into diverse thermal management applications. In addition, the latent heat and encapsulation ratio values obtained for the microPCMs make them suitable for latent heat energy storage (LHTES) applications, including passive solar space heating, thereby reinforcing their relevance for sustainable building and energy systems [60].

Table 2 presents a comparative analysis of the thermal and encapsulation performance of microencapsulated phase change materials (MEPCMs) incorporating various shell materials and core–shell configurations. This comprehensive study assesses the thermal characteristics and encapsulation effectiveness of different MEPCM formulations, including paraffin-based systems with inorganic shells (CaCO_3_ and CaCl_2_), polymer shells (PS and MF), and matrices modified with graphene oxide. According to the study, beeswax/CaCl_2_ formulations achieve highly competitive encapsulation ratios (83.43–88.93%) with moderate latent heat values (119–121 kJ/kg), making them viable options for thermal energy storage applications at moderate temperatures. The results of the study demonstrate how well shell materials and nanofillers work together to improve thermal stability and heat storage capacity, paving the way for more sophisticated thermal energy storage applications in a variety of temperature ranges.

## 4. Conclusions

This study successfully synthesized and thoroughly characterized innovative microencapsulated phase change materials (BW@CaCO_3_ microPCMs), presenting a sustainable and efficient solution for thermal energy storage applications. By employing beeswax (BW) as the bio-based PCM core and calcium carbonate (CaCO_3_) as the shell material, an eco-friendly approach was achieved without compromising performance.

Structural analyses (FTIR, XRD, and SEM) confirmed the formation of well-defined spherical microcapsules with strong core–shell integrity. The CaCO_3_ shell, composed of vaterite and calcite phases, provided enhanced thermal stability and mechanical robustness.

Differential scanning calorimetry (DSC) revealed that a shell content of approximately 40% offers the best balance between energy storage capacity and structural stability. This formulation exhibited high latent heat, reduced supercooling, and excellent reproducibility during repeated thermal cycling. The synergistic interaction between BW and CaCO_3_ effectively addresses two critical limitations of conventional PCMs—thermal instability and supercooling—thereby improving reliability for practical applications.

Overall, BW@CaCO_3_ microPCMs represent a promising, sustainable, and high-performance material for advanced thermal energy storage systems, paving the way for their integration into energy-efficient building materials and other green technologies.

Nevertheless, some limitations remain. Thermal cycling assessment relied primarily on qualitative DSC curve analysis, preventing precise quantification of enthalpy variations across cycles. Moreover, the encapsulation process may require further optimization to minimize shell agglomeration and enhance microcapsule uniformity, which could influence long-term durability.

## Figures and Tables

**Table 1:** Phase change behavior and performance of beeswax and BW@CaCO_3_.

Samples	T_fusion_ (°C)	ΔHf (J/g)	Er (%) Experimental	Er (%) Calculated
Beeswax	58.30	137.62	-	-
microPCM20%	58.06	122.22	88.08	85.62
microPCM40%	56.49	121.39	88.93	74.78
microPCM60%	57.63	120.42	87.50	66.63
microPCM80%	57.38	119.81	83.43	59.81

**Table 2:** Comparative thermal and encapsulation performance of microencapsulated phase change materials.

MEPCMs	Er (%)	T_fusion_ (°C)	ΔHf (J/g)	Ref.
Paraffin@CaCO_3_ (SDBS-7)	68.8	51.3	177.5	[47]
Paraffin@CaCO_3_ (SDS-7)	62.1	56.0	160.2	[47]
Paraffin@CaCO_3_ (SMA-7)	56.6	58.3	146.0	[47]
1.0 EG/MEPCM	86.33	44.48	181.3	[61]
1.0 GO/MEPCM	87.67	44.63	184.1	[61]
1.5 Mn GO/MEPCM	88.28	43.86	185.4	[61]
paraffin@PS	78.5	-	148.5	[62]
MicroPCM/EG (30 wt%)	58.23	48.46	95.48	[63]
MicroPCM/EG (20 wt%)	35.77	48.71	113.9	[63]
RT 28@CaCO_3_	-	26.57	179.3	[64]
RT 42@CaCO_3_	-	47.36	238.2	[64]
palmitic acid (PA)@TiO_2_ MPCM1	30.4	-	63.3	[65]
palmitic acid (PA)@TiO_2_ MPCM2	11.1	60.7	23.2	[65]
palmitic acid (PA)@TiO_2_ MPCM3	15.9	60.7	33.1	[65]
n-Tetradecane	93.2	-	195.9	[66]
n-Eicosane/NPZ mass ratio (50/50)	61.98	43.75	123.4	[67]
n-Eicosane/NPZ mass ratio (40/60)	52.43	43.38	103.8	[67]
n-Octadecane/CaCl_2_ mass ratio (30/70)	21.89	28.09	46.93	[68]
n-Octadecane/CaCl_2_ mass ratio (40/60)	32.04	28.22	67.91	[68]
n-Octadecane/CaCl_2_ mass ratio (50/50)	40.04	29.19	84.37	[68]
Beeswax/CaCl_2_ mass ratio (60/40)	88.93	56.5	121.39	This study
Beeswax/CaCl_2_ mass ratio (40/60)	87.5	57.6	120.42	This study
Beeswax/CaCl_2_ mass ratio (80/20)	83.43	57.4	119.81	This study
Beeswax/CaCl_2_ mass ratio (60/40)	88.93	56.5	121.39	This study

## Data Availability

The original contributions presented in this study are included in the article. Further inquiries can be directed to the corresponding authors.

## Abbreviations

SDS	Sodium dodecyl sulfate
BW	Beeswax
microPCMs	Microencapsulated phase change materials
PCMs	Phase change materials
SEM-EDS	Scanning electron microscopy and energy-dispersive spectrometer
FTIR	Fourier transform infrared spectroscopy
XRD	X-ray diffractometer
DSC	Differential scanning calorimetry
